# Comparison of ^18^F-AIF-NOTA-PRGD2 and ^18^F-FDG Uptake in Lymph Node Metastasis of Differentiated Thyroid Cancer

**DOI:** 10.1371/journal.pone.0100521

**Published:** 2014-06-23

**Authors:** Weiwei Cheng, Zhenyu Wu, Sheng Liang, Hongliang Fu, Shuqi Wu, Yiyun Tang, Zhiyi Ye, Hui Wang

**Affiliations:** Department of Nuclear Medicine, Xin Hua Hospital Affiliated to Shanghai Jiao Tong University School of Medicine, Shanghai, China; Wayne State University, United States of America

## Abstract

A widespread application of integrin α_v_β_3_ imaging has been emerging in both pre-clinical and clinical studies. But few studies reported its value as compared with ^18^F-FDG PET, especially for differentiated thyroid cancer (DTC). In this study, we compared the tracer uptake of ^18^F-AIF-NOTA-PRGD2 and ^18^F-FDG in lymph node metastasis of DTC to evaluate ^18^F-AIF-NOTA-PRGD2 as compared with ^18^F-FDG.

**Methods:**

20 DTC patients with presumptive lymph node metastasis were examined with ^18^F-AIF-NOTA-PRGD2 and ^18^F-FDG PET/CT. 16 patients undergoing fine needle aspiration biopsy (FNAB) were evaluated by cytology results. For lesions without FNAB, the findings of clinical staging procedures served as the standard of reference (including neck ultrasound and serum thyroglobulin).

**Results:**

A total of 39 presumptive lymph node metastases were visualized on PET/CT images. 35 lesions were confirmed as malignant by FNAB and other clinical findings. The mean ^18^F-AIF-NOTA-PRGD2 in radioactive iodine-refractory (RAIR) lesions and benign lesions were 2.5±0.9 and 2.8±0.9 respectively. The mean SUV for ^18^F-FDG in all malignant lesions was 4.5±1.6 while in benign lesions it was 3.3±1.2. For all malignant lesions, the mean SUV for ^18^F-FDG was significantly higher than that for ^18^F-AIF-NOTA-PRGD2 (P<0.05). No significant correlation was found between the SUVs of ^18^F-AIF-NOTA-PRGD2 and ^18^F-FDG for 35 lesions (r = 0.114, *P* = 0.515). Moreover, 15 lesions of which the diameter larger than 1.5cm had higher ^18^F-AIF-NOTA-PRGD2 uptake as compared with the lesions smaller than 1.5cm.

**Conclusion:**

Although most lymph node metastases of DTC showed abnormal uptake of ^18^F-AIF-NOTA-PRGD2, its diagnostic value was inferior to ^18^F-FDG. No correlation was found between the uptake of ^18^F-AIF-NOTA-PRGD2 and ^18^F-FDG, which may suggest the two tracers provide complementary information in DTC lesions.

## Introduction

Integrin α_v_β_3_ receptor has been widely studied and was found to play essential roles in angiogenesis and tumor metastasis. It expressed preferentially on various tumor cells and endothelial cells but was low on mature endothelial and epithelial cells [1]–[5]. Nowadays, integrin α_v_β_3_ has been considered a valuable target for diagnosis and therapy of malignant tumors [6]–[7]. Labeled ligands bearing the peptide of Arg-Gly-Asp (RGD) have a high affinity and specificity for integrin α_v_β_3_, and have been applied for integrin α_v_β_3_ imaging in both pre-clinical and clinical studies [8]–[9]. Recently, for the first time Zhao et al. applied RGD imaging in differentiated thyroid carcinoma (DTC) patients and they concluded that RGD imaging was a promising modality for diagnosing and guiding further treatment of radioactive iodine-refractory (RAIR) DTC [10].

As we all know, for DTC patients with RAIR lesions, ^18^F-FDG PET/CT was generally recommended to localize lesions by American Thyroid Association [11]. Although Zhao et al. has reported that all RAIR lesions presented higher uptake of the radiolabeled RGD tracer, it has not evaluated how the RGD tracer behaved in comparison with the common tumor diagnostic agent ^18^F-FDG. This is of great importance, since RGD imaging can not only localize RAIR lesions but also can plan further targeted therapy for RAIR DTC while ^18^F-FDG PET/CT is a diagnosis-only modality. Therefore, we want to know whether there is a possibility that RGD imaging can replace ^18^F-FDG PET/CT to be applied to RAIR DTC lesions. Up till now, no study reported such comparison in DTC lesions.

Moreover, since both integrin α_v_β_3_ expression and increased glucose metabolism are believed to correlate with tumor aggressiveness and prognosis [12]–[13], we cannot exclude that there is a correlation between the RGD tracer uptake and ^18^F-FDG uptake, which means ^18^F-FDG may provide information similar to that of ^18^F-AIF-NOTA-PRGD2 although the two tracers have completely different pharmacological mechanism. In case of a close correlation between the uptakes of the two tracers, there would be no need for the new RGD imaging agents, as ^18^F-FDG has already successfully applied in clinical routine.

In this study, we evaluated the integrin α_v_β_3_ expression of lymph node metastases in DTC by using a novel RGD peptide tracer ^18^F-AIF-NOTA-PRGD2 [14]–[15], and compared the uptake of ^18^F-AIF-NOTA-PRGD2 and ^18^F-FDG in lymph node metastases of DTC to evaluate RGD imaging in DTC patients and whether there was a correlation between the two tracers' uptake.

## Patients and Methods

20 DTC patients (11 women and 9 men) were recruited from the Nuclear Medicine Department of Shanghai Xin Hua Hospital. The mean age was 38.6±16.2 y (rang: 21–65 y) and the median weight was 71.5 kg (rang: 43–132 kg). All patients had undergone a previous total or near-total thyroidectomy and radioiodine ablation therapy. Patient inclusion was based on the following criteria: age over 18 y; a negative pregnancy test; clinically acceptable renal and hepatic function; patients were suspected with lymph node metastasis which based on neck ultrasound (US) and serum thyroglobulin (TG), with additional information from computed tomography (CT) and diagnostic ^131^I-whole body scan (^131^I-WBS). Before the ^131^I-WBS and PET/CT imaging, patient' serum TSH were controlled to 30 mU/L. 16 patients were scheduled to undergo US-guided fine-needle aspiration biopsy (FNAB) within 3 weeks after PET/CT imaging. For lesions without FNAB as the inappropriate locations and 4 patients who didn't want to perform with FNAB, other clinical findings (including neck US, serum TG and TG-antibody) and clinical follow-up served as the standard of reference.

### ^18^F-AIF-NOTA-PRGD2 PET/CT

PET/CT imaging was performed on a Biograph 64 PET/CT scanner (Siemens Biograph MCT) in all instances. One hour after the intravenous injection of ^18^F-AIF-NOTA-PRGD2 (201.4±45.1 MBq; range, 138.4–347.8 MBq) to the patients, the successive whole-body PET/CT scans were obtained [14]. The lowest possible milliampere setting on the scanner was used to acquire the CT scans for attenuation correction. The helical CT scan acquisition parameters were 120 kV, 0.5 s rotation; 3 mm slice thickness, and 0.8 mm interval. The PET scans were acquired in 3-dimensional mode and ranged from the top of the head to mid thigh. The scan time was 1.5 min per bed position, and each scan covered 7 bed positions, with single-slice overlap between the bed positions.

### ^18^F-FDG PET/CT

Standard patient preparation before ^18^F-FDG PET/CT imaging included at least six hours of fasting and a serum glucose level of less than 6.7 mmol/L before tracer injection. Each patient received 259–407 MBq (7–11 mCi) ^18^F-FDG intravenously. After tracer injection, the patients rested on a comfortable chair during the ^18^F-FDG uptake period. PET/CT was initiated 60 min after injection of ^18^F-FDG.

### Imaging Analysis

The acquired images were analyzed with Analysis Software (Medex). The tracer uptake was expressed in standardized uptake values (SUVs), which was calculated according to the following formula: (measured activity concentration [Bq/ml]×body weight [g])/injected activity [Bq]. An ellipsoidal volume of interest (VOI) was drawn around the highest uptake to include the entire lesion. The outer border of each VOI was semiautomatically defined by an isocontour representing 60% of the maximum activity within the VOI. The mean SUV in this VOI was used for further analysis. For lesions that were not identifiable on the image of one tracer (^18^F-AIF-NOTA-PRGD2 or ^18^F-FDG), the VOI was placed at the site of the lesions according to CT and the image of the other tracer. The major axis of lymph nodes in CT was used for analysis. To avoid a bias by patients with an exceptionally high number of lesions (n>4), the lesions with the highest tracer uptake were chosen (4 lesions per person).

### Statistics Analysis

All quantitative data were expressed as mean ± SD. Wilcoxon Test was performed to compare the SUVs between malignant and benign lesions. The correlation between quantitative parameters was evaluated by linear regression analysis and by calculation of the Pearson correlation coefficient R. Statistical significance was tested using SPSS (Version 19.0, IBM) at the level of 5%.

### Ethics Statement

The study was approved by the ethics committee of the Shanghai Jiao Tong University and Shanghai Xin Hua Hospital and by the institutional review boards of Shanghai Xinhua Hospital. Informed written consents were obtained from all patients.

## Results

20 DTC patients with a total of 39 presumptive lymph node metastases were visualized on PET/CT images, including 33 RAIR lesions and 6 iodine-avid lesions. FNAB were carried out on 20 RAIR lesions and 5 iodine-avid lesions. The results showed 21 lesions were malignant and 4 lesions were benign. For four patients who did not perform FNAB, a total of nine RAIR lesions were found. All the nine lesions plus the five other lesions (without performing FNAB since the inappropriate location) were confirmed as metastases by the other clinical findings including abnormal neck US images, elevated serum TG (median: 94.3 ng/L, rang: 1–638.4 ng/L) and serum TG-antibody (for the only patient with normal TG, the serum TG-antibody was 68.75 u/mL). In brief, 35 lesions were malignant and 4 lesions were benign. Further details of the patients were presented in Table 1.

**Table 1:** Patients' characteristics.

				SUVmean			
Patient no.	Gender	Age(y)	Size of lymph node (major * minor axis)	^18^F-AIF-NOTA-PRGD2	^18^F-FDG	Lesions undergo FNAB*	Malignant or benign^#^
1	F	35	18*10	3.1	2.9	-	+
			21*16	3.1	6.9	+	+
2	F	26	9*7	2.6	5.6	+	+
			16*11	3.2	4.7	+	-
			11*10	2.2	4.0	+	+
			11*10	1.8	4.2	+	+
3	M	21	8*4	1.6	2.5	+	-
4	F	45	19*12	2.8	2.6	+	+
			18*17	2.8	2.2	+	+
			14*11	4.2	4.4	-	+
			10*8	3.5	4.3	+	+
5	M	65	19*17	3.9	8.0	+	+
			16*13	3.1	5.7	+	+
			16*10	3.2	6.4	+	+
			17*9	3.0	5.0	-	+
6	F	22	14*10	3.8	3.8	+	-
7	F	60	15*14	3.9	2.8	+	+
8	M	59	16*15	2.8	4.0	+	+
9	M	27	13*7	2.5	2.2	+	-
10	F	26	27*22	4.1	3.8	+	+
			14*11	2.7	2.9	+	+
11	M	33	16*11	2.8	3.3	+	+
12	M	35	16*13	4.0	5.3	+	+
			11*10	2.8	5.0	-	+
			9*9	2.7	6.7	+	+
13	M	21	10*6	1.2	3.8	-	+
14	F	49	11*10	2.2	5.6	+	+
15	M	41	12*7	1.4	3.7	-	+
16	M	24	8*7	3.7	3.4	+	+
			11*8	2.9	4.3	-	+
17	F	38	15*11	1.7	6.6	-	+
			13*10	1.9	4.5	-	+
			12*7	1.8	4.0	-	+
18	F	58	8*6	0.4	2.8	-	+
			7*6	1.5	3.4	-	+
			13*9	2.1	5.4	-	+
			11*7	2.2	4.3	-	+
19	F	50	17*10	1.6	8.2	+	+
20	F	38	11*10	1.4	2.8	+	+

*:+:performed with FNAB;-:without performing FNAB.

#:+:malignant;-:benign.

**Figure 1:**
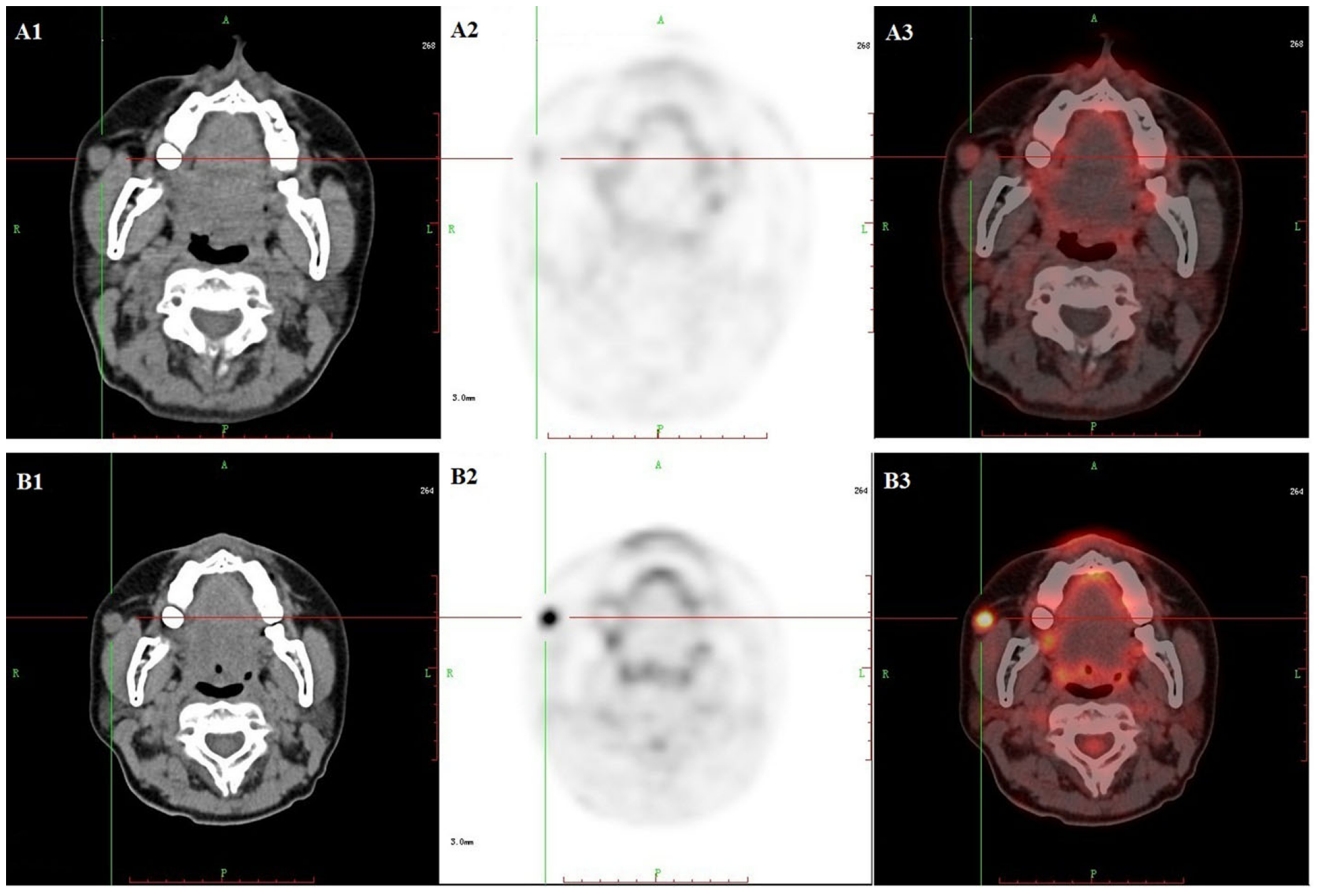
Comparison of different uptake patterns in ^18^F-AIF-NOTA-PRGD2 PET and ^18^F-FDG PET. The enlarged lymph node identified on CT (A1 and B1) had significantly higher uptake of ^18^F-FDG PET (B2–B3) than that for ^18^F-AIF-NOTA-PRGD2 (A2–A3).

**Figure 2:**
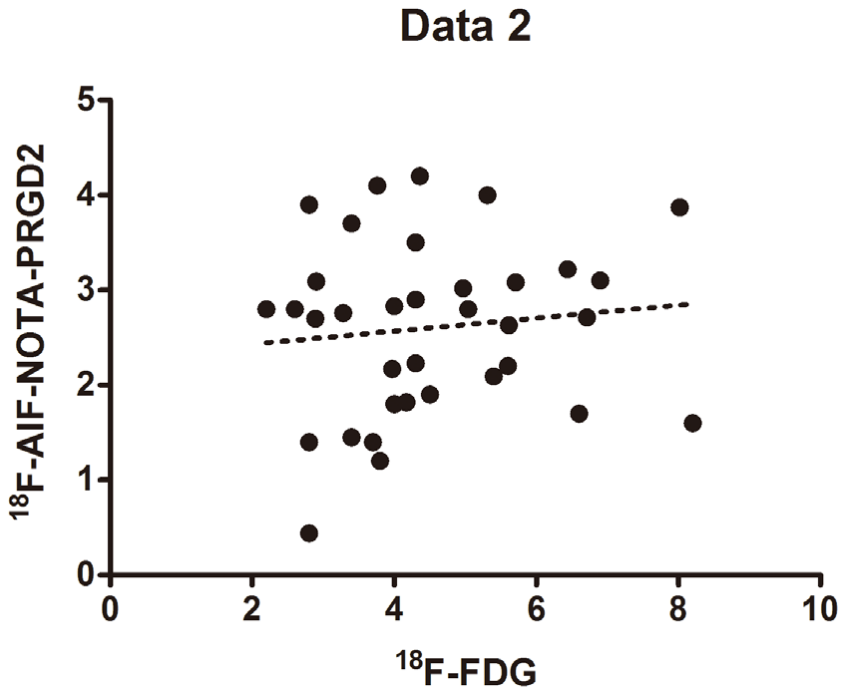
Comparison of SUVs from ^18^F-FDG PET and ^18^F-AIF-NOTA-PRGD2 PET for all malignant lesions.

The mean SUVs for ^18^F-AIF-NOTA-PRGD2 in all RAIR lesions (n = 29) and all benign lesions (n = 4) were 2.5±0.9 and 2.8±0.9 respectively. The mean SUV for ^18^F-FDG in all RAIR lesions was 4.5±1.6 while in benign lesions it was 3.3±1.2. Neither ^18^F-AIF-NOTA-PRGD2 nor ^18^F-FDG showed significantly different tracer uptake in malignant and benign lesions (P = 0.576 and 0.133, respectively). Further studies were necessary since the small number of benign lesions in this study. For all malignant lesions, the mean SUV for ^18^F-FDG was significantly higher than that for ^18^F-AIF-NOTA-PRGD2 (P<0.05) (Figure 1). The SUVs of ^18^F-AIF-NOTA-PRGD2 in 5 lesions was even lower than 1.5 while ^18^F-FDG uptake was significant (median: 3.7; rang: 3.2–8.2). There was only one lesion in which the mean SUV of ^18^F-FDG was lower than 2.5, whereas showed higher ^18^F-AIF-NOTA-PRGD2 uptake (mean SUV = 2.8). No significant correlation was found between the SUVs of ^18^F-AIF-NOTA-PRGD2 and ^18^F-FDG for 35 malignant lesions (r = 0.114, *P* = 0.515; Fig.2).

There were 25 lymph nodes of which the diameter was larger than 1 cm. No statistically differences of mean SUVs of ^18^F-AIF-NOTA-PRGD2 and ^18^F-FDG had been found between the lesions larger than 1 cm and the lesions smaller than 1 cm (P = 0.06 and 0.41, respectively). However, 15 lesions of which the diameter larger than 1.5 cm had slightly higher uptake of ^18^F-AIF-NOTA-PRGD2 as compared with the lesions smaller than 1.5 cm (3.1±0.7vs 2.4±0.8; P<0.05) while there was still no significant difference of ^18^F-FDG uptake between different sizes of lymph node lesions.

Three patients (Patient No. 10–12) in our study with six lesions in total concentrated radioiodine. The SUVs of ^18^F-AIF-NOTA-PRGD2 in these six lesions were among 2.7–4.0 (mean: 3.2). We compared the mean SUVs of RAIR (n = 29) and iodine-avid lesions (n = 6). The result showed that no significant difference of ^18^F-AIF-NOTA-PRGD2 uptake was found as well as ^18^F-FDG (P>0.05).

## Discussion

Noninvasive PET imaging of integrin α_v_β_3_ has become an important tool for tumor diagnosis and treatment monitoring in both preclinical and clinical studies [6]-[7], [14]. Currently, several radiolabeled RGD peptides have been evaluated in clinical trials, and a more widespread use of PET imaging of integrin expression is expected in the near future.

In terms of diagnosis, a multicenter study of ^99^mTc-3PRGD2 for integrin receptor imaging of lung cancer indicated that ^99^mTc-3PRGD2 imaging at 1 h was sensitive enough for the detection of lung cancer, with a sensitivity of 88% for semiquantitative analysis [9]. Also, the study conducted by Zhao et al. revealed that RGD imaging is valuable in diagnosis of RAIR DTC [10]. As the widespread application of PET/CT in China and its relatively high resolution, for the first time we applied a new PET imaging agent ^18^F-AIF-NOTA-PRGD2 in DTC patients, which showed longer tumor retention and simpler labeling process, and has been successfully used in lung cancer patients and myocardial infarction/reperfusion animal model [14]–[15]. As we know, the absence of iodine-concentration ability of RAIR DTC lesions posed a relatively difficulty in lesion location and therapy [16]. Therefore, the majority of patients involved in our study were RAIR DTC lesions. In this study, we compared ^18^F-AIF-NOTA-PRGD2 PET/CT with ^18^F-FDG PET/CT, to see whether the former can replace the later in terms of its diagnostic value in RAIR DTC lesions, with its additional superiority of assessing tumor angiogenesis.

The mean SUV for ^18^F-AIF-NOTA-PRGD2 of all malignant lesions in this study was 2.6±0.9, while that for ^18^F-FDG was 4.5±1.5 (P<0.05). Although the two tracers have totally different pharmacodynamic mechanisms, it is a fact that a higher SUV usually increases the chance of getting a reliable diagnosis. In most cases, the SUV of 2.5 was considered as the cut-off value in our clinical routine, with a combination of other considerations in practical diagnosis. In our study, SUVs for RGD tracer in many malignant lesions are just around this cut-off value and some even lower than 1.5, which can result in low sensitivity. Some studies attributed the lower SUV for RGD tracer to the reason that ^18^F-FDG accumulated in the larger numbers of tumor cells whereas ^18^F-labeled RGD peptide bound mainly to the smaller number of endothelial cells [17]. However, although no study has reported integrin α_v_β_3_ expression in DTC lesions, there were studies have observed that RGD peptide not only bound to tumor endothelial cells but also tumor cells and no significant difference in binding were found between the two kinds of cells [18].

Furthermore, five benign lymph node lesions having been confirmed as inflammatory hyperplasia by FNAB also showed a high SUV value (2.8±0.9). This is not surprising, since integrin α_v_β_3_ plays an essential role not only in tumor progression but also in macrophage inflammatory responses [19]. This means ^18^F-AIF-NOTA-PRGD2 PET/CT may have a low specificity, which need further studies involving larger samples to confirm. Cervical lymph nodes with inflammation could also show high ^18^F-FDG uptake on PET, but in overall ^18^F-FDG PET/CT was reported to be 71% sensitive, 96% specific and 81.3% accurate for identification of RAIR DTC lesions [20].

Nevertheless, since the primary intention of integrin α_v_β_3_ imaging was not meant to diagnose tumors but rather to evaluate tumor angiogenesis, it has a promising value in identifying potential therapeutic target and monitoring anti-angiogenesis therapy[7]. For DTC patients with RAIR lesions, as we know, surgical resection and external beam radiotherapy represent the only therapeutic options. Chemotherapy was usually not effective since the lack of enrollment of patients with therapeutic targets [21]. RAIR DTC lesions are normally regarded to be more aggressive and metastatic [22], revealing cells are more likely to have high integrin α_v_β_3_ expression, which can be targeted by integrin α_v_β_3_ inhibitors. From the results of our study, some RAIR lesions did show a high integrin α_v_β_3_ expression while others did not. In this sense, ^18^F-AIF-NOTA-PRGD2 PET can provide evaluation of tumor angiogenesis for planning and monitoring of target anti-angiogenesis therapies for RAIR DTC patients. Moreover, in prostate cancer, brain tumor and tumors showed low ^18^F-FDG uptake [23]–[25], ^18^F-AIF-NOTA-PRGD2 PET may still have advantages in terms of diagnosis.

As we mentioned above, integrin α_v_β_3_ expression and glucose metabolism were both believed to correlate with tumor aggressiveness and progression. Therefore, some link was assumed to exist between ^18^F-AIF-NOTA-PRGD2 and ^18^F-FDG despite their completely different pharmacodynamic mechanisms. Actually, many reports have described a correlation between ^18^F-FDG uptake and angiogenesis in vitro and in vivo [12]–[13]. Cheng et al. have demonstrated that ^18^F-FDG can be used to monitor the treatment of the integrin inhibitor cilengitide which targets α_v_β_3_ and α_v_β_5_ receptors in bone metastasis [26]. In our study, however, no such correlation was found between the mean SUVs of ^18^F-AIF-NOTA-PRGD2 and that of ^18^F-FDG, which meant two tracers provide complementary information in DTC lesions. Our result was in line with some other studies, which believed that ^18^F-FDG uptake was independent of angiogenesis [27]. Since no correlation between ^18^F-FDG uptake and angiogenesis was found in DTC lesions, ^18^F-FDG PET/CT can't replace RGD imaging in terms of evaluating tumor angiogenesis and planning α_v_β_3_ therapy.

Additionally, we investigated the mean SUVs of lymph nodes with different sizes. As we know, when tumors grow beyond 2–3mm, the increased interstitial pressure within the tumor inhibits the diffusion of metabolites and nutrients necessary for tumor growth and a state of cellular hypoxia begins, which can lead to tumor angiogenesis [28]–[29]. Moreover, Zhao's study has demonstrated a correlation between rapid tumor growth and integrin α_v_β_3_ expression in RAIR lesions [10]. Based on these findings, a positive correlation between integrin α_v_β_3_ expression and lesion size would have been conceivable. In our study, in lesions larger than 1.5 cm, the mean SUV of ^18^F-AIF-NOTA-PRGD2 was higher than lesions smaller than 1.5 cm. This result partly showed a positive correlation between integrin α_v_β_3_ expression and lesion size. Nevertheless, we must consider the impact of the partial-volume effect which lowers the SUV value of the small size lesion and occurs typically when the lesion size is less than 1 cm [30].

Apart from RAIR DTC lesions, we also include three patients with six iodine-avid DTC lesions. The SUVs of ^18^F-AIF-NOTA-PRGD2 iodine-avid lesions were among 2.7-4.0 (mean: 3.2) and no significant difference of tracer uptake was found between RAIR and iodine-avid lesions. This result may indicate that these lesions have a potential of invasion and metastatic and require more aggressive treatments. However, since in our study patients with iodine-avid lesions were arranged to have radioiodine ablation therapy, such high integrin α_v_β_3_ expression may attribute to ablation-induced injury and repair and the small number of iodine-avid lesions was also likely to lead to inconclusive results.

## Conclusion

Although most lymph node metastases of DTC showed abnormal uptake of ^18^F-AIF-NOTA-PRGD2, its diagnostic value was inferior to the traditional imaging agent ^18^F-FDG while it had an advantage in evaluation of planning and monitoring targeted anti-angiogenesis therapy. No correlation was found between the uptake of ^18^F-AIF-NOTA-PRGD2 and ^18^F-FDG, suggesting the two tracers provided complementary information in DTC lesions.
